# *FGFR 1*–*3* Alteration Frequency in Muscle-Invasive Urothelial Bladder Cancer and Its Effect on Prognosis

**DOI:** 10.3390/medicina62102026

**Published:** 2026-10-20

**Authors:** Dilek Çağlayan, Emine Göktaş, Makbule Nihan Somuncu, Fahriye Kılınç, Mehmet Zahid Koçak, Engin Hendem, Oğuzhan Yıldız, Ali Fuat Gürbüz, Hilal Canlar, Muzaffer Uğraklı, Melek Karakurt Eryılmaz, Mehmet Artaç, Murat Araz

**Affiliations:** 1 Department of Medical Oncology, Faculty of Medicine, Necmettin Erbakan University, Konya,42090, Turkey; mehmetzahidkocak@hotmail.com (M.Z.K.); dr.oguzhan@outlook.com (O.Y.); dr.alifuatg@gmail.com (A.F.G.); mugrakli@gmail.com (M.U.); drangelkarakurt@hotmail.com (M.K.E.); mehmetartac@yahoo.com (M.A.); zaratarum@yahoo.com (M.A.); 2 Department of Medical Genetics, Faculty of Medicine, Necmettin Erbakan University, Konya 42090, Turkey; emineaktas88@hotmail.com (E.G.); mnsomuncu@gmail.com (M.N.S.); 3 Department of Pathology, Faculty of Medicine, Necmettin Erbakan University, Konya 42090, Turkey; drfahriyek@gmail.com; 4 Department of Medical Oncology, Mengücek Gazi Training and Research Hospital, Erzincan 24100, Turkey; enginhende61@gmail.com; 5 Department of Internal Medicine, Faculty of Medicine, Necmettin Erbakan University, Konya 42090, Turkey; hilaltiryaki2101@gmail.com

**Keywords:** muscle-invasive bladder cancer, *FGFR* alterations, prognosis

## Abstract

*Background and Objectives:* Fibroblast growth factor receptor (*FGFR*) alterations are therapeutic targets in urothelial carcinoma, but their prognostic significance in muscle-invasive bladder cancer (MIBC) is uncertain. We evaluated the frequency of *FGFR1–3* alterations and their association with overall survival (OS) in Turkish patients with MIBC. *Materials and Methods:* Formalin-fixed, paraffin-embedded tumour samples from 194 patients with non-metastatic muscle-invasive (n = 154) or metastatic (n = 40) urothelial carcinoma of the bladder were analysed by targeted next-generation sequencing of *FGFR1–3*. Variants were classified according to ACMG/AMP tiers. OS was assessed with Kaplan–Meier and Cox regression analyses in the overall cohort and in both subgroups. *Results:* At least one *FGFR* alteration was detected in 131 patients (67.5%): *FGFR3* in 42.8%, *FGFR1* in 24.7% and *FGFR2* in 20.6%. Most *FGFR1* (91.7%) and *FGFR2* (87.5%) alterations were Tier III variants, whereas Tier I *FGFR3* hotspot mutations were found in 17.5% of the cohort. Median OS was shorter in *FGFR*-altered than in *FGFR*-unaltered patients (14 vs. 18 months; *p* = 0.024), with a similar difference in the non-metastatic subgroup (19 vs. 24 months; *p* = 0.047) but not in the metastatic subgroup (8 vs. 7 months; *p* = 0.653). After adjustment for metastatic status, ECOG performance status and age, overall *FGFR* alteration status was not an independent prognostic factor (HR 1.31, 95% CI 0.87–1.97; *p* = 0.200), whereas *FGFR1* alteration was independently associated with worse OS (HR 1.62, 95% CI 1.07–2.44; *p* = 0.023). *FGFR3* alterations were not prognostic. *Conclusions: FGFR* alterations, predominantly in *FGFR3*, are frequent in MIBC and are associated with shorter OS, but not independently of metastatic status and performance status. *FGFR1* alteration was associated with poor prognosis, but this finding requires validation in larger cohorts.

## 1. Introduction

Bladder cancer is the ninth most common cancer worldwide, and GLOBOCAN 2022 estimates that there are 615,000 new cases diagnosed annually [1]. Multiple genetic and environmental factors are involved in the development of bladder cancer. Bladder cancer is associated with factors such as hereditary cancer syndromes, exposure to carcinogenic chemical compounds, smoking and infections [2,3,4,5,6]. In general, urothelial bladder cancer is differentiated into two groups: non-muscle-invasive bladder cancer and muscle-invasive bladder cancer. At the time of diagnosis, 75% of bladder cancers are non-muscle-invasive, and 25% are muscle-invasive or metastatic [7]. Non-muscle invasive bladder cancers are mainly characterized by activation of the *RAS/MAPK* pathway by the *FGFR3* pathway, whereas muscle invasive bladder cancers are characterized by alterations in the retinoblastoma (Rb) and p53 pathways regulating the cell cycle [8,9].

Fibroblast growth factor receptors are a family of tyrosine kinases that consist of 4 different receptors (*FGFR1–4*) that have extracellular, transmembrane, and intracellular domains [10,11,12]. *FGFR*s regulate cell proliferation, survival, migration and differentiation. Mutations and fusions of *FGFR 2* and *3* are frequent in patients with urothelial cancer. These mutations and fusions may activate *FGFR* signaling and promote carcinogenesis [13].

Activating mutations of *FGFR3* are often caused by point mutations or gene fusions, and are among the most common genetic alterations in urothelial cancer [14,15]. They are found in 70% of nonmuscle invasive bladder cancers and 15% of muscle invasive bladder cancers [15,16,17]. They represent targets for targeted therapies.

In phase 2 trials with the pan-*FGFR* inhibitor erdafitinib, an objective response rate of 40% was achieved in patients with *FGFR* driver mutations, with a median duration of response of 5.6 months [18].

In this study, we aimed to evaluate the frequency of *FGFR 1–2–3* alterations and their effect on prognosis in metastatic and non-metastatic bladder cancer.

## 2. Material and Methods

### 2.1. Study Design and Clinical Samples

This was a retrospective cohort study in which molecular analysis was performed on archived tumour tissue samples after ethical approval. Adults (≥18 years) diagnosed with locally advanced muscle-invasive or metastatic urothelial carcinoma of the bladder and followed between 1 January 2015 and 19 May 2023 were screened. Of 294 patients, 81 were excluded because archived tumor blocks were unavailable and 19 because tumor cellularity or DNA quality was inadequate for next-generation sequencing (NGS), leaving 194 evaluable patients. FFPE tumor blocks were identified from the pathology archives according to the eligibility criteria; following ethical approval, DNA was extracted, and *FGFR1*, *FGFR2*, and *FGFR3* sequencing analyses were performed. Demographic, clinicopathological, and treatment data were obtained from institutional records. The study was conducted according to the Declaration of Helsinki and was approved by the institutional ethics committee of the institution in 2023 (No. 2023/4343).

### 2.2. DNA Extraction and Quality Control

Genomic DNA was isolated from formalin-fixed, paraffin-embedded tumor tissue using the QIAamp DNA FFPE Advanced Kit (QIAGEN, Hilden, Germany; cat. no. 56604) according to the manufacturer’s instructions and stored at −20 °C until analysis. DNA concentration was measured using a Qubit fluorometer(Thermo Fisher Scientific, Waltham, MA, USA), with NanoDrop spectrophotometry (Thermo Fisher Scientific, Waltham, MA, USA) performed when required. Only samples meeting the input and quality criteria of the target-capture workflow proceeded to library preparation.

### 2.3. Targeted NGS and Bioinformatic Analysis

A custom-designed hybridization-based target-capture panel (Celemics, Inc., Seoul, Republic of Korea) was used to analyze *FGFR1*, *FGFR2*, and *FGFR3*. DNA was enzymatically fragmented to approximately 150–250 bp, followed by magnetic-bead purification, end repair, A-tailing, adapter ligation, dual indexing, and library amplification. Indexed libraries were hybridized overnight at 65 °C with *FGFR1–3* capture probes, recovered using streptavidin-coated beads, amplified by post-capture PCR, purified, quantified, pooled, and sequenced on a MiSeq platform (Illumina, Inc., San Diego, CA, USA). Demultiplexed reads were exported in FASTQ format.

FASTQ files were analyzed using the SEQ Platform (Genomize Inc., Istanbul, Turkey) and aligned to the hg19 human reference genome. Sequencing data were considered analytically adequate when >95% of the targeted regions were achieved. A minimum read variant allele frequency (VAF) threshold of 5% was applied to Tier III variants, whereas a threshold of 1% was used for all other evaluated variants.

Following alignment to the designated human reference genome and variant calling, reportable *FGFR1–3* variants were described according to Human Genome Variation Society nomenclature. Franklin by Genoox (https://franklin.genoox.com, accessed on 29 September 2026) and VarSome (Saphetor SA, Lausanne, Switzerland; https://varsome.com, accessed on 29 September 2026) were used for integrated annotation and evidence review; COSMIC, CIViC, and OncoKB were queried for prior cancer observations, oncogenicity, clinical significance, and potential diagnostic, prognostic, or therapeutic relevance. Evidence was evaluated collectively across these resources. Somatic variants were classified according to the American College of Medical Genetics and Genomics/Association for Molecular Pathology (ACMG/AMP) criteria using a tier-based clinical-significance framework (Tier I, strong clinical significance; Tier II, potential clinical significance; Tier III, unknown clinical significance; Tier IV, benign/likely benign) [19,20]. Patients with at least one reportable finding were categorized as *FGFR*-altered; those without a reportable *FGFR1–3* finding were categorized as *FGFR*-unaltered.

### 2.4. Outcomes

Molecular outcomes were the frequency, spectrum, and tier distribution of *FGFR1*, *FGFR2*, and *FGFR3* alterations. The clinical outcome was overall survival, evaluated in the full cohort and separately in non-metastatic and metastatic subgroups.

### 2.5. Statistical Analysis

Overall survival (OS) was defined as the time from diagnosis to death from any cause; patients alive at last follow-up were censored. OS was estimated with the Kaplan–Meier method, 95% confidence intervals (CIs) for median OS were calculated with the Brookmeyer–Crowley method, and groups were compared with the log-rank test. Median follow-up was estimated with the reverse Kaplan–Meier method. Survival analyses were performed in the overall cohort and separately in the non-metastatic and metastatic subgroups. Hazard ratios (HRs) were estimated with Cox proportional hazards regression. Two prespecified multivariable models were constructed: Model A included overall *FGFR* alteration status, and Model B included *FGFR1*, *FGFR2* and *FGFR3* alteration status simultaneously, so that the effect of each gene was adjusted for co-occurring alterations in the other genes. Both models were adjusted for metastatic status, ECOG performance status and age in the overall cohort, and for ECOG performance status, primary treatment modality and age in the non-metastatic subgroup; *FGFR* variables were retained in the models irrespective of their univariable significance. Stage was not included because stage IV was collinear with metastatic status. In the metastatic subgroup, only exploratory ECOG performance status-adjusted models were fitted because of the limited number of events. Effect modification by metastatic status was assessed by adding an *FGFR* × metastatic status interaction term (likelihood-ratio test). A sensitivity analysis restricted *FGFR*-altered status to Tier I/II variants. The proportional hazards assumption was verified with scaled Schoenfeld residuals. Patients with missing ECOG performance status were excluded from the multivariable models (complete-case analysis). Categorical variables were compared with the chi-square or Fisher’s exact test and continuous variables with the Mann–Whitney U test. A two-sided *p* < 0.05 was considered statistically significant. Analyses were performed with IBM SPSS Statistics version 25 (IBM Corp., Armonk, NY, USA) and Python version 3.11 (Python Software Foundation) using the lifelines (v0.30.3) and statsmodels (v0.15.0) packages. 

## 3. Results

### 3.1. Patient Characteristics

**Figure 1:**
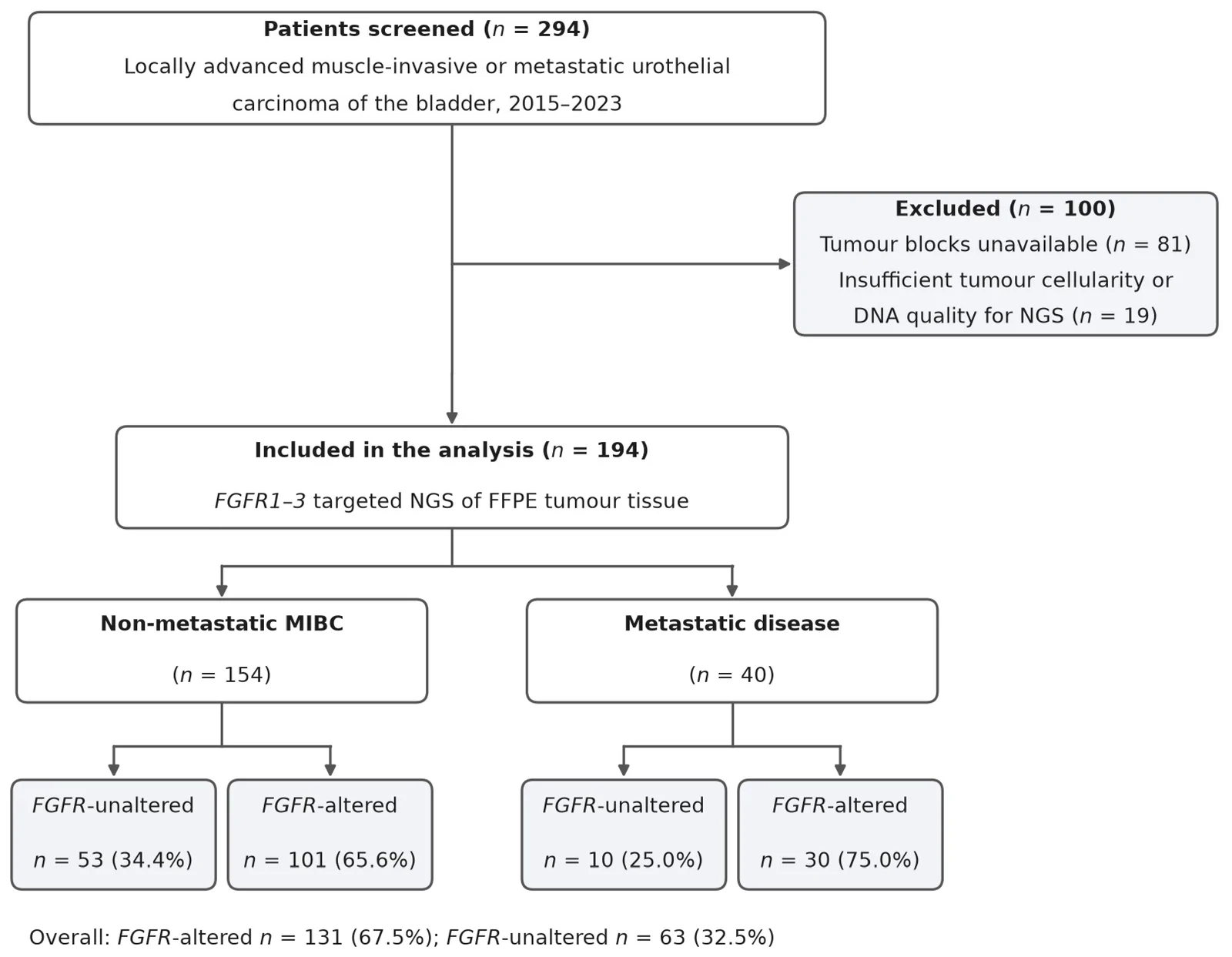
Study Flowchart.

Of the 294 patients screened, 194 with evaluable NGS results were included in the analysis (Figure 1); 154 (79.4%) had non-metastatic muscle-invasive disease and 40 (20.6%) had metastatic disease at diagnosis. At least one *FGFR* alteration was detected in 101 of 154 (65.6%) non-metastatic patients and in 30 of 40 (75.0%) metastatic patients (*p* = 0.34).

Baseline characteristics according to *FGFR* alteration status within each subgroup are summarised in Table 1. In the non-metastatic subgroup, *FGFR*-altered patients were numerically older than *FGFR*-unaltered patients (median age 70 vs. 66 years; *p* = 0.11), but there were no significant differences in sex, smoking status, alcohol use, family history of cancer, ECOG performance status (PS), comorbidities, prior transurethral resection, stage or primary treatment (all *p* ≥ 0.15). Of the 154 non-metastatic patients, 32 (20.8%) received neoadjuvant chemotherapy followed by radical cystectomy, 33 (21.4%) underwent surgery followed by adjuvant chemotherapy, 34 (22.1%) received chemoradiotherapy, and 55 (35.7%) did not receive curative-intent treatment. In the metastatic subgroup, the two groups were also balanced; 29 of 40 patients (72.5%) received first-line systemic therapy (90.0% of *FGFR*-unaltered vs. 66.7% of *FGFR*-altered patients; *p* = 0.23). ECOG PS was not recorded in 49 patients (25.3%).

### 3.2. Frequency and Spectrum of FGFR Alterations

At least one *FGFR* alteration was identified in 131 of 194 patients (67.5%). *FGFR3* was the most frequently altered gene (83 patients, 42.8%), followed by *FGFR1* (48 patients, 24.7%) and *FGFR2* (40 patients, 20.6%). The tier distribution differed markedly between genes (Table 2). Tier III variants accounted for 91.7% of *FGFR1* and 87.5% of *FGFR2* alterations, and no Tier I variant was found in either gene. In contrast, 34 of 83 *FGFR3*-altered patients (41.0%) harboured a Tier I variant, corresponding to 17.5% (34/194) of the entire cohort; a further 15 (18.1%) had Tier II and 34 (41.0%) Tier III variants. All *FGFR3* Tier I variants were recurrent activating hotspot mutations: p.Ser249Cys (n = 21), p.Arg248Cys (n = 8), p.Tyr373Cys (n = 3) and p.Gly370Cys (n = 2).

At the variant level, 204 variants were identified (*FGFR1*, 64; *FGFR2*, 48; *FGFR3*, 92). Missense substitutions were the predominant variant type in all three genes (161/204, 78.9%), followed by splice-region/intronic variants (n = 19), nonsense variants (n = 16), in-frame insertions/deletions (n = 5) and frameshift variants (n = 3). Truncating (nonsense or frameshift) variants were observed mainly in *FGFR1* (12/64 variants) and *FGFR2* (6/48), whereas only one was found in *FGFR3* (Table 2). All detected alterations were single-nucleotide variants or small insertions/deletions; gene fusions and copy-number alterations were not assessed by the panel.

Alterations in more than one *FGFR* gene were common (Table 3). Among the 131 *FGFR*-altered patients, 93 (71.0%) had an alteration in a single gene, 32 (24.4%) in two genes and 6 (4.6%) in all three genes. An isolated *FGFR3* alteration was the most frequent pattern (n = 60), followed by isolated *FGFR1* (n = 19) and *FGFR2* (n = 14) alterations. Patients were classified as *FGFR*-altered if at least one reportable variant of any tier was present in *FGFR1*, *FGFR2* or *FGFR3*. For gene-level analyses, each gene was coded as a separate binary variable regardless of alterations in the other genes, and co-occurring alterations were accounted for by entering all three genes simultaneously into the multivariable model.

### 3.3. Survival According to FGFR Alteration Status

At a median follow-up of 88 months (reverse Kaplan–Meier method), 161 patients (83.0%) had died. Median OS was 15 months (95% CI 12–18) in the overall cohort, 20 months (95% CI 15–25) in the non-metastatic subgroup and 8 months (95% CI 5–9) in the metastatic subgroup.

**Figure 2:**
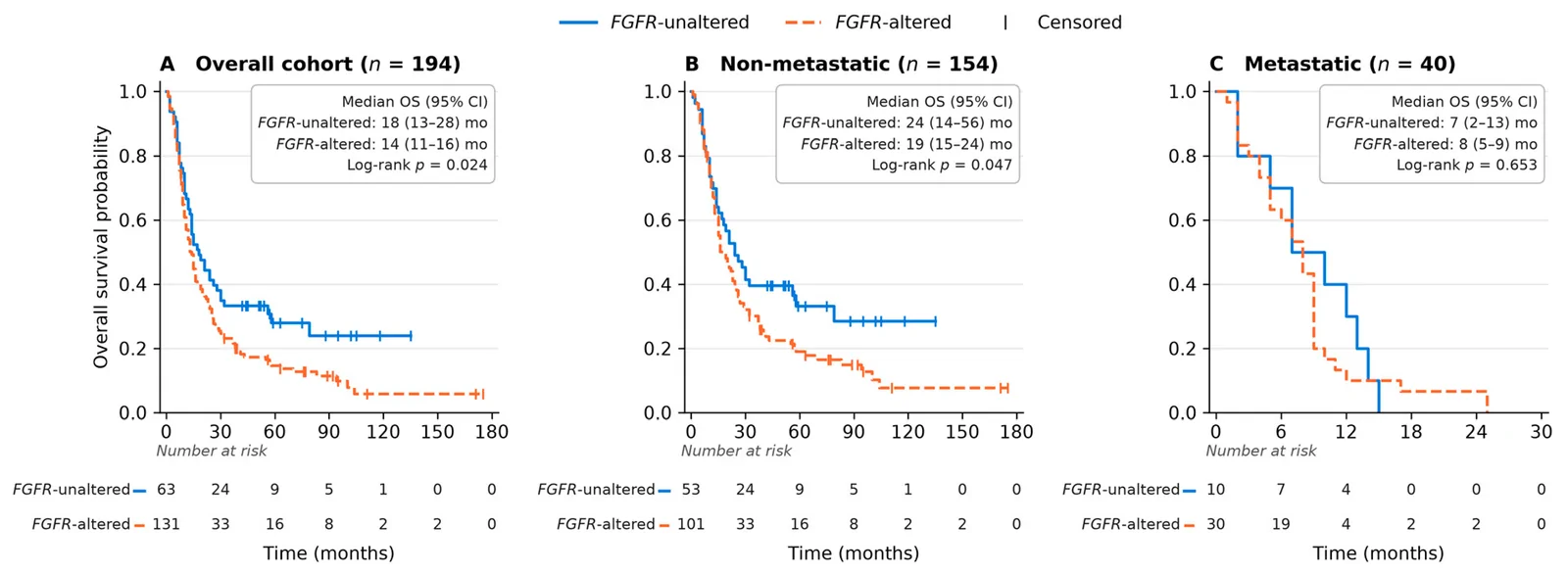
Kaplan–Meier estimates of overall survival by *FGFR* alteration status in (**A**) the overall cohort (n = 194), (**B**) the non-metastatic subgroup (n = 154) and (**C**) the metastatic subgroup (n = 40). Solid blue lines, *FGFR*-unaltered; dashed orange lines, *FGFR*-altered; vertical ticks, censored observations. Median overall survival with 95% confidence intervals (Brookmeyer–Crowley method) and two-sided log-rank *p* values are shown in each panel; numbers at risk are given below the x-axis. Overall cohort: 18 months (95% CI 13–28) vs. 14 months (95% CI 11–16), *p* = 0.024. Non-metastatic: 24 months (95% CI 14–56) vs. 19 months (95% CI 15–24), *p* = 0.047. Metastatic: 7 months (95% CI 2–13) vs. 8 months (95% CI 5–9), *p* = 0.653. CI, confidence interval; NR, not reached; OS, overall survival.

In the overall cohort, median OS was shorter in *FGFR*-altered than in *FGFR*-unaltered patients (14 months [95% CI 11–16] vs. 18 months [95% CI 13–28]; log-rank *p* = 0.024; Figure 2A). A similar difference was observed in the non-metastatic subgroup (19 months [95% CI 15–24] vs. 24 months [95% CI 14–56]; *p* = 0.047; Figure 2B), whereas no difference was found in the metastatic subgroup (8 months [95% CI 5–9] vs. 7 months [95% CI 2–13]; *p* = 0.653; Figure 2C).

**Figure 3:**
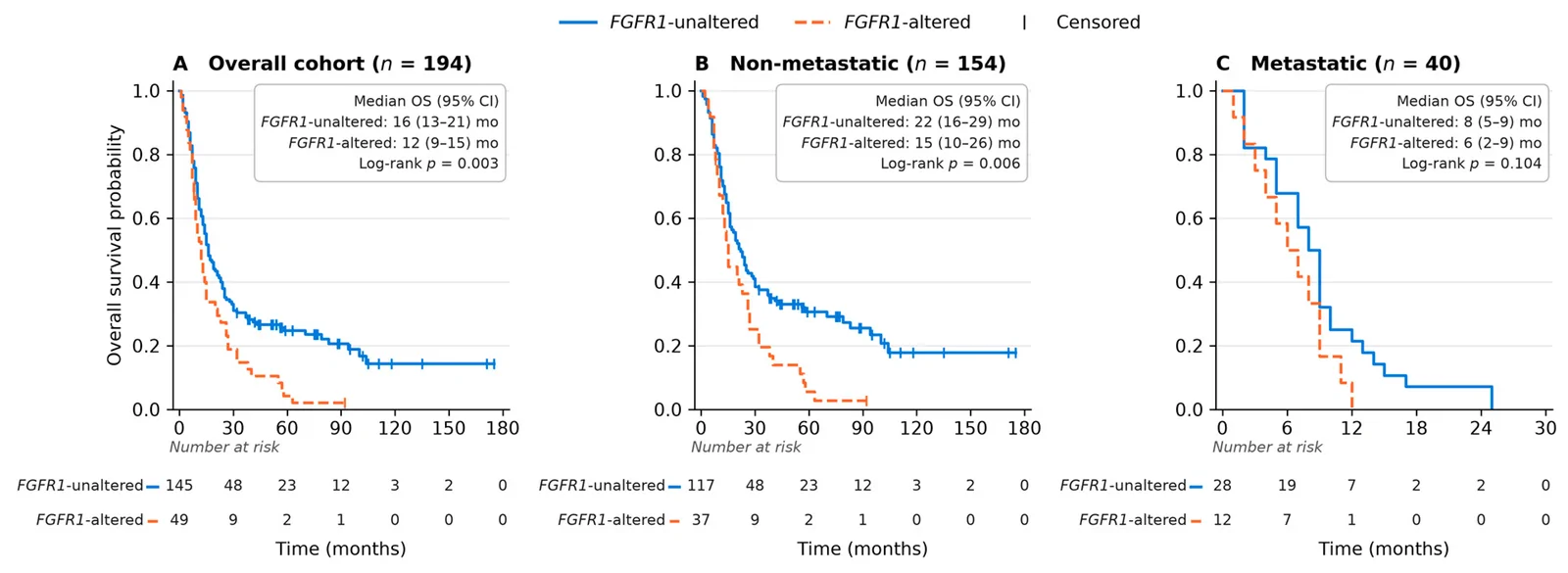
Kaplan–Meier estimates of overall survival by *FGFR1* alteration status in (**A**) the overall cohort (n = 194), (**B**) the non-metastatic subgroup (n = 154) and (**C**) the metastatic subgroup (n = 40). Solid blue lines, *FGFR1*-unaltered; dashed orange lines, *FGFR1*-altered; vertical ticks, censored observations. Median overall survival with 95% confidence intervals (Brookmeyer–Crowley method) and two-sided log-rank *p* values are shown in each panel; numbers at risk are given below the x-axis. Overall cohort: 16 months (95% CI 13–21) vs. 12 months (95% CI 9–15), *p* = 0.003. Non-metastatic: 22 months (95% CI 16–29) vs. 15 months (95% CI 10–26), *p* = 0.006. Metastatic: 8 months (95% CI 5–9) vs. 6 months (95% CI 2–9), *p* = 0.104. CI, confidence interval; NR, not reached; OS, overall survival.

*FGFR1*-altered patients had shorter OS than *FGFR1*-unaltered patients in the overall cohort (12 months [95% CI 9–15] vs. 16 months [95% CI 13–21]; *p* = 0.003; Figure 3A) and in the non-metastatic subgroup (15 months [95% CI 10–26] vs. 22 months [95% CI 16–29]; *p* = 0.006; Figure 3B). In the metastatic subgroup, the difference was not significant (6 months [95% CI 2–9] vs. 8 months [95% CI 5–9]; *p* = 0.104; Figure 3C).

**Figure 4:**
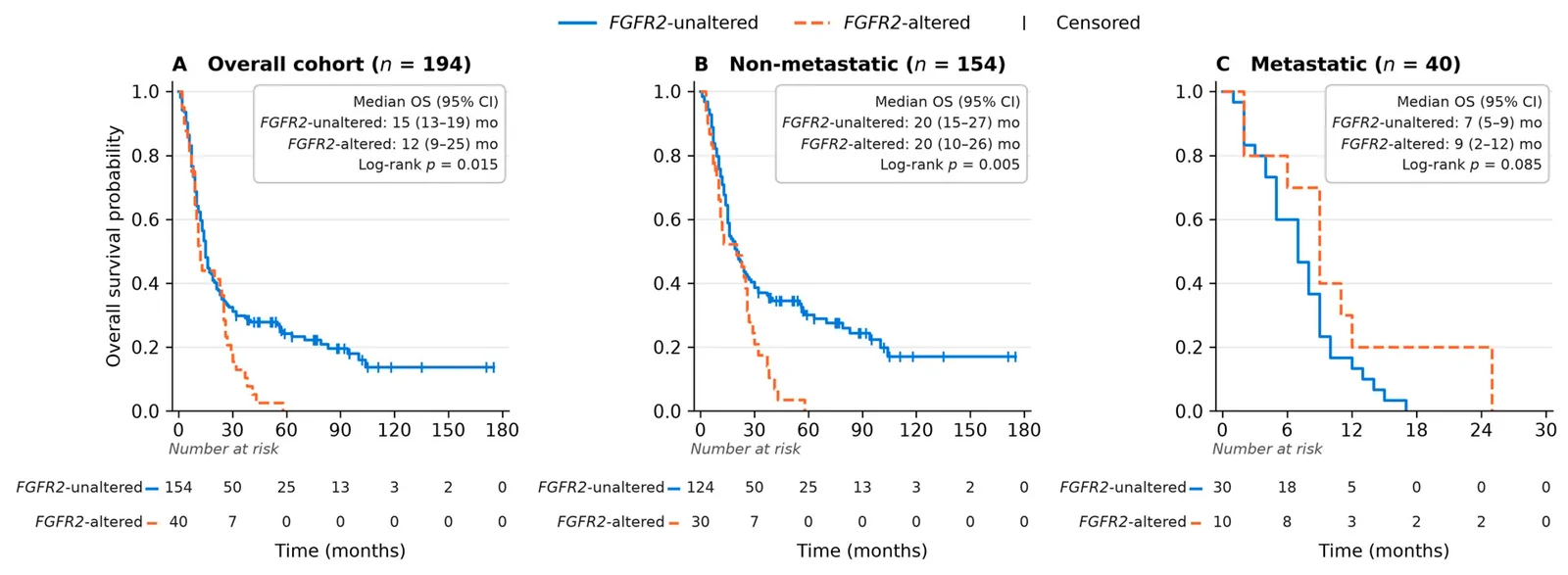
Kaplan–Meier estimates of overall survival by *FGFR2* alteration status in (**A**) the overall cohort (n = 194), (**B**) the non-metastatic subgroup (n = 154) and (**C**) the metastatic subgroup (n = 40). Solid blue lines, *FGFR2*-unaltered; dashed orange lines, *FGFR2*-altered; vertical ticks, censored observations. Median overall survival with 95% confidence intervals (Brookmeyer–Crowley method) and two-sided log-rank *p* values are shown in each panel; numbers at risk are given below the x-axis. Overall cohort: 15 months (95% CI 13–19) vs. 12 months (95% CI 9–25), *p* = 0.015. Non-metastatic: 20 months (95% CI 15–27) vs. 20 months (95% CI 10–26), *p* = 0.005. Metastatic: 7 months (95% CI 5–9) vs. 9 months (95% CI 2–12), *p* = 0.085. CI, confidence interval; NR, not reached; OS, overall survival.

*FGFR2* alteration was associated with shorter OS in the overall cohort (12 months [95% CI 9–25] vs. 15 months [95% CI 13–19]; *p* = 0.015; Figure 4A). In the non-metastatic subgroup, the log-rank test was significant (*p* = 0.005), although median OS was identical (20 vs. 20 months) and the curves crossed, with the difference emerging only beyond approximately 24 months (Figure 4B). No difference was seen in the metastatic subgroup (9 vs. 7 months; *p* = 0.085; Figure 4C).

**Figure 5:**
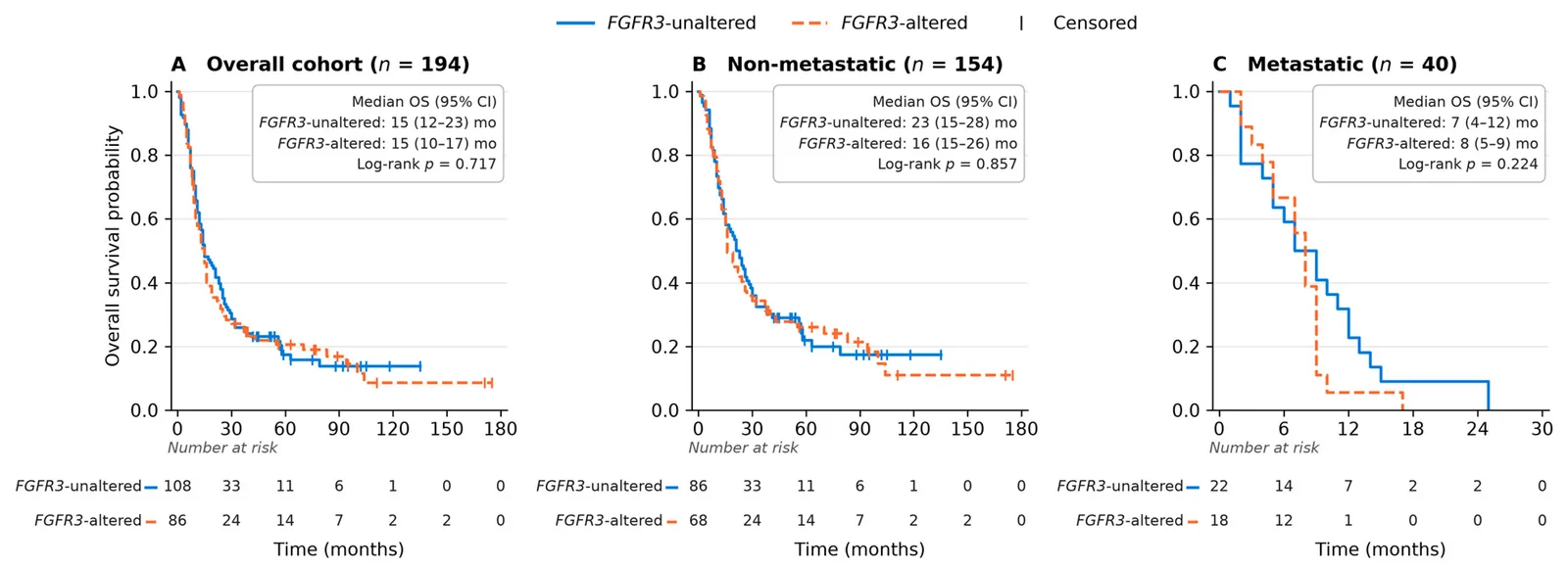
Kaplan–Meier estimates of overall survival by *FGFR3* alteration status in (**A**) the overall cohort (n = 194), (**B**) the non-metastatic subgroup (n = 154) and (**C**) the metastatic subgroup (n = 40). Solid blue lines, *FGFR3*-unaltered; dashed orange lines, *FGFR3*-altered; vertical ticks, censored observations. Median overall survival with 95% confidence intervals (Brookmeyer–Crowley method) and two-sided log-rank *p* values are shown in each panel; numbers at risk are given below the x-axis. Overall cohort: 15 months (95% CI 12–23) vs. 15 months (95% CI 10–17), *p* = 0.717. Non-metastatic: 23 months (95% CI 15–28) vs. 16 months (95% CI 15–26), *p* = 0.857. Metastatic: 7 months (95% CI 4–12) vs. 8 months (95% CI 5–9), *p* = 0.224. CI, confidence interval; NR, not reached; OS, overall survival.

*FGFR3* alteration was not associated with OS in the overall cohort (15 months [95% CI 10–17] vs. 15 months [95% CI 12–23]; *p* = 0.717; Figure 5A), the non-metastatic subgroup (16 vs. 23 months; *p* = 0.857; Figure 5B) or the metastatic subgroup (8 vs. 7 months; *p* = 0.224; Figure 5C).

### 3.4. Univariable Cox Regression Analysis

Univariable Cox regression results are shown in Table 4. In the overall cohort, metastatic disease (HR 4.30, 95% CI 2.90–6.38; *p* < 0.001), older age (HR 1.32 per 10 years, 95% CI 1.12–1.54; *p* <0.001) and ECOG PS 2 (HR 2.38, 95% CI 1.34–4.25; *p* = 0.003) were associated with worse OS. Any *FGFR* alteration (HR 1.47, 95% CI 1.04–2.08; *p* = 0.028), *FGFR1* alteration (HR 1.65, 95% CI 1.17–2.32; *p* = 0.004) and *FGFR2* alteration (HR 1.56, 95% CI 1.08–2.25; *p* = 0.018) were also associated with shorter OS, whereas *FGFR3* alteration was not (HR 1.06, 95% CI 0.77–1.44; *p* = 0.723). Neither *FGFR3* Tier I alteration (HR 0.74, 95% CI 0.48–1.12; *p* = 0.155) nor the presence of any Tier I/II *FGFR* alteration (HR 1.02, 95% CI 0.73–1.43; *p* = 0.903) was associated with OS.

In the non-metastatic subgroup, any *FGFR* alteration showed a borderline association with worse OS (HR 1.48, 95% CI 1.00–2.19; Wald *p* = 0.052; likelihood-ratio *p* = 0.046), while *FGFR1* (HR 1.72, 95% CI 1.15–2.56; *p* = 0.008) and *FGFR2* (HR 1.81, 95% CI 1.18–2.76; *p* = 0.007) alterations were significantly associated with worse OS. *FGFR3* Tier I alteration showed a non-significant trend towards longer OS (HR 0.61, 95% CI 0.36–1.02; *p* = 0.060). In the metastatic subgroup, receipt of first-line systemic therapy was the only factor associated with OS (HR 0.26, 95% CI 0.12–0.57; *p* <0.001); none of the *FGFR* variables was significant.

### 3.5. Multivariable Cox Regression Analysis

Multivariable analyses are presented in Table 5. In the overall cohort (n = 145 with complete data; 119 deaths), no *FGFR* alteration was independently associated with OS after adjustment for metastatic status, ECOG PS and age (Model A: HR 1.31, 95% CI 0.87–1.97; *p* = 0.200). At the gene level (Model B), *FGFR1* alteration remained an independent predictor of worse OS (HR 1.62, 95% CI 1.07–2.44; *p* = 0.023), whereas *FGFR2* (HR 1.05, 95% CI 0.66–1.65; *p* = 0.841) and *FGFR3* (HR 1.25, 95% CI 0.86–1.81; *p* = 0.242) alterations were not. Metastatic disease (HR 5.34, 95% CI 3.23–8.86; *p* < 0.001) and age (HR 1.32 per 10 years, 95% CI 1.06–1.63; *p* = 0.012) were also independent predictors. The prognostic effect of *FGFR* alteration did not differ by metastatic status (interaction *p* = 0.59).

In the non-metastatic subgroup (n = 115; 89 deaths), after adjustment for ECOG PS, primary treatment and age, neither any *FGFR* alteration (Model A: HR 1.41, 95% CI 0.88–2.26; *p* = 0.154) nor individual *FGFR1* (HR 1.56, 95% CI 0.95–2.57; *p* = 0.081), *FGFR2* (HR 1.56, 95% CI 0.93–2.60; *p* = 0.091) or *FGFR3* (HR 1.12, 95% CI 0.71–1.76; *p* = 0.635) alterations were independently associated with OS, although the direction and magnitude of the *FGFR1* effect were consistent with the overall cohort. In the metastatic subgroup, exploratory ECOG PS-adjusted models (30 deaths) showed no significant association between any *FGFR* variable and OS.

In a sensitivity analysis restricting *FGFR*-altered status to Tier I/II variants, no independent association with OS was observed (HR 1.36, 95% CI 0.90–2.04; *p* = 0.141). The proportional hazards assumption was satisfied for all models (scaled Schoenfeld residuals, all *p* > 0.05).

## 4. Discussion

In this single-centre cohort of 194 patients with muscle-invasive or metastatic urothelial carcinoma of the bladder, at least one *FGFR1–3* alteration was identified in two-thirds of patients. *FGFR3* was the most frequently altered gene and the only gene harboring Tier I hotspot mutations, whereas the great majority of *FGFR1* and *FGFR2* alterations were Tier III variants of uncertain clinical significance. *FGFR* alterations as a group were associated with shorter OS in univariable analyses, but this association was not independent of metastatic status, performance status and age. At the gene level, *FGFR1* alteration remained independently associated with worse OS in the overall cohort, whereas *FGFR3* alteration was not prognostic in any setting.

*FGFR3* Tier I alterations were detected in 34 patients (17.5%). Most Tier I variants were concentrated in hotspot regions in exon 6 of chromosome 4. The most frequently identified mutation was the *Ser249Cys* missense mutation. These results were consistent with those reported in the literature [15,16,17]. While Tier I alterations were detected only in *FGFR3*, no Tier I variants were identified in *FGFR1* or *FGFR2*. Among patients with *FGFR1* alterations, 8.3% harbored Tier II, while the remaining patients had Tier III variants. Among patients with *FGFR2* alterations, 12.5% harbored Tier II variants, while the remaining patients had Tier III variants. Among patients with *FGFR3* alterations, 41% had Tier I variants, 18.1% had Tier II variants, and 41% had Tier III variants.

In the literature, *FGFR1* and *FGFR3* alterations are frequently identified in bladder cancer and play a role in its pathogenesis. *FGFR2* alterations are rare and have a limited role in bladder cancer pathogenesis. *FGFR4* alterations have been reported in almost no cases [21,22,23].

The frequency of *FGFR3* alterations decreases with increasing stage and grade. It is observed in 65% of papillary tumors and 12% of muscle-invasive tumors. The frequency is 70% in grade 1 tumors and 19% in grade 3 tumors [24,25,26]. Although *FGFR3* alterations are common in low-grade and non-invasive disease, a study conducted in patients with metastatic disease demonstrated that the presence of *FGFR* genomic alterations was associated with poor prognosis [27].

In a meta-analysis evaluating 15 studies, data from 1963 patients with urothelial carcinoma were assessed. Patients in this meta-analysis had been treated with immunotherapy at any line of treatment. Of these patients, 21.6% were *FGFR*-altered, and 78.4% were *FGFR* wild-type. Pooled quantitative results demonstrated that *FGFR*-altered patients had worse overall survival compared to *FGFR* wild-type patients (HR: 1.23, 95% CI: 1.04–1.44, *p* = 0.01). In addition, *FGFR* alteration was associated with lower disease control rates following treatment with immune checkpoint inhibitors (OR: 0.65, 95% CI: 0.43–0.97, *p* = 0.04). *FGFR*-altered patients had worse progression-free survival (*p* = 0.02) and similar disease-specific survival (*p* = 0.11) compared to *FGFR-unaltered* patients [28]. The immunosuppressive tumor microenvironment in *FGFR*-altered urothelial carcinomas may explain the reduced response to immunotherapy [29]. However, the effect of *FGFR3* alterations on response to immunotherapy remains controversial. In a real-world cohort of patients with metastatic urothelial carcinoma, which included patients with poor performance status and adverse prognostic features who are often excluded from clinical trials, *FGFR3*-altered and *FGFR3*-unaltered tumours showed comparable responses to immune checkpoint inhibitors [30]

Although the response to immune checkpoint inhibitors remains controversial, it has been shown that in patients with metastatic urothelial carcinoma harboring an *FGFR3* alteration, treatment with erdafitinib—a pan-*FGFR* inhibitor—following immune checkpoint inhibitor therapy prolongs survival compared with chemotherapy [31].

Although many recent studies have focused on the role of *FGFR3* in bladder cancer, very little is known about the expression and effects of other members of the *FGFR* family in the development of bladder cancer. A study examining the transcript levels of *FGFR1–4* demonstrated that *FGFR1* expression is elevated in a large proportion of bladder cancer cell lines and in most tumors, regardless of stage or grade. This finding suggests that increased *FGFR1* expression represents an early stage in the development of bladder cancer [32].

In our study, screening of the *FGFR* panel *(FGFR1–3)* in muscle-invasive bladder cancer revealed a high proportion of Tier III variant accumulation. While Tier I variants in *FGFR3* (particularly hotspot regions such as S249C) are well established in the literature, the fact that the majority of variants in *FGFR1* and *FGFR2* are of Tier III status reflects the complex and yet unmapped roles of these genes in the pathogenesis of muscle-invasive bladder cancer (MIBC). The loss of independent prognostic value of overall *FGFR* alteration status in multivariate analysis may be attributable to the biological heterogeneity introduced by this high Tier III burden.

The limitations of our study include its retrospective design, the limited sample size, and the fact that patients with driver mutations did not receive erdafitinib due to reimbursement restrictions. Among patients with metastatic disease, the majority received platinum-based chemotherapy, whereas only one patient received immunotherapy due to local reimbursement restrictions. Owing to the limited sample size within these treatment subgroups, subgroup-specific Kaplan–Meier survival analyses stratified by treatment modality could not be performed.

The strengths of our study include the use of NGS for *FGFR1–3* alteration analysis from patient tumor tissue, and the evaluation of the prognostic impact of *FGFR* alterations not only in metastatic disease but also in non-metastatic muscle-invasive bladder cancer patients treated with local therapies.

## 5. Conclusions

*FGFR* alterations, predominantly in *FGFR3*, are frequent in MIBC and are associated with shorter OS, although this association is not independent of metastatic status and performance status. *FGFR1* alteration was associated with poor prognosis; however, as most *FGFR1* variants were of uncertain clinical significance, this finding should be regarded as hypothesis-generating and needs to be validated in larger cohorts.

## Figures and Tables

**Table 1:** Baseline characteristics of patients by *FGFR* alteration status in the non-metastatic and metastatic subgroups.

Characteristic	Non-Metastatic (n = 154)			Metastatic (n = 40)		
	*FGFR*-Unaltered (n = 53)	*FGFR*-Altered (n = 101)	*p*	*FGFR*-Unaltered (n = 10)	*FGFR*-Altered (n = 30)	*p*
Age, years, median (IQR)	66 (59–73)	70 (64–76)	0.11	66 (58–71)	70 (60–75)	0.34
Male sex	44 (83.0)	90 (89.1)	0.32	6 (60.0)	24 (80.0)	0.23
**Smoking status**						
Never	22 (41.5)	27 (26.7)	0.15	5 (50.0)	8 (26.7)	0.45
Former	13 (24.5)	29 (28.7)		3 (30.0)	12 (40.0)	
Current	9 (17.0)	26 (25.7)		1 (10.0)	5 (16.7)	
Unknown	9 (17.0)	19 (18.8)		1 (10.0)	5 (16.7)	
**Alcohol use**						
Yes	4 (7.5)	6 (5.9)	0.74	0 (0.0)	0 (0.0)	
No	42 (79.2)	78 (77.2)		9 (90.0)	24 (80.0)	
Unknown	7 (13.2)	17 (16.8)		1 (10.0)	6 (20.0)	
**Family history of cancer**						
Yes	12 (22.6)	16 (15.8)	0.37	2 (20.0)	3 (10.0)	1.00
No	32 (60.4)	66 (65.3)		8 (80.0)	17 (56.7)	
Unknown	9 (17.0)	19 (18.8)		0 (0.0)	10 (33.3)	
**ECOG performance status**						
0	8 (15.1)	20 (19.8)	0.81	2 (20.0)	2 (6.7)	0.36
1	24 (45.3)	44 (43.6)		3 (30.0)	15 (50.0)	
2	6 (11.3)	13 (12.9)		2 (20.0)	6 (20.0)	
Missing	15 (28.3)	24 (23.8)		3 (30.0)	7 (23.3)	
**Comorbidities**						
Any comorbidity	27 (50.9)	61 (60.4)	0.30	7 (70.0)	20 (66.7)	1.00
Diabetes mellitus	9 (17.0)	17 (16.8)	1.00	2 (20.0)	10 (33.3)	0.69
Hypertension	8 (15.1)	12 (11.9)	0.62	1 (10.0)	2 (6.7)	1.00
Coronary artery disease	5 (9.4)	11 (10.9)	1.00	1 (10.0)	5 (16.7)	1.00
COPD/asthma	3 (5.7)	9 (8.9)	0.55	1 (10.0)	1 (3.3)	0.44
Chronic kidney disease	3 (5.7)	3 (3.0)	0.41	0 (0.0)	0 (0.0)	
Other	9 (17.0)	28 (27.7)	0.17	3 (30.0)	6 (20.0)	0.66
Prior TURBT	47 (88.7)	90 (89.1)	1.00	8 (80.0)	25 (83.3)	1.00
**Stage at diagnosis**						
II	17 (32.1)	38 (37.6)	0.60	–	–	
III	36 (67.9)	63 (62.4)		–	–	
IV	–	–		10 (100.0)	30 (100.0)	
**Primary treatment (non-metastatic)**						
Neoadjuvant CT + surgery	9 (17.0)	23 (22.8)	0.48	–	–	
Surgery + adjuvant CT	15 (28.3)	18 (17.8)		–	–	
Chemoradiotherapy	11 (20.8)	23 (22.8)		–	–	
No curative-intent therapy	18 (34.0)	37 (36.6)		–	–	
**Systemic therapy (metastatic)**						
First-line systemic therapy	–	–		9 (90.0)	20 (66.7)	0.23
Immunotherapy/enfortumab vedotin (any line)	–	–		1 (10.0)	0 (0.0)	0.25

Values are n (%) unless otherwise stated. *p* values compare *FGFR*-unaltered vs. *FGFR*-altered patients within each subgroup (Fisher’s exact or chi-square test for categorical variables, excluding unknown/missing categories; Mann–Whitney U test for age). CT, chemotherapy; ECOG, Eastern Cooperative Oncology Group; COPD, chronic obstructive pulmonary disease; IQR, interquartile range; TURBT, transurethral resection of bladder tumour.

**Table 2:** Tier classification and molecular consequence of *FGFR1–3* variants.

Gene	Patients with Tiered Variant, n	Tier I, n (%)	Tier II, n (%)	Tier III, n (%)
*FGFR1*	48	0	4 (8.3)	44 (91.7)
*FGFR2*	40	0	5 (12.5)	35 (87.5)
*FGFR3*	83	34 (41.0)	15 (18.1)	34 (41.0)
**Variant Consequence (Variant Level)**		***FGFR1***	***FGFR2***	***FGFR3***
Missense		42	34	85
Nonsense		10	5	1
Frameshift		2	1	0
In-frame indel		2	2	1
Splice region/intronic		8	6	5
Total variants		64	48	92

Percentages are of patients with a tiered variant in the respective gene. Variants were classified according to the AMP/ASCO/CAP joint consensus recommendation: Tier I, strong clinical significance; Tier II, potential clinical significance; Tier III, unknown clinical significance. Counts are of individual variants; some patients carried more than one variant in the same gene. All alterations detected were single-nucleotide variants or small insertions/deletions. *FGFR3* Tier I variants: p.Ser249Cys (n = 21), p.Arg248Cys (n = 8), p.Tyr373Cys (n = 3), p.Gly370Cys (n = 2).

**Table 3:** Co-occurrence of *FGFR1*, *FGFR2* and *FGFR3* alterations.

Alteration Pattern	Non-Metastatic (n = 154)	Metastatic (n = 40)	Total (n = 194)
*FGFR1* only	15	4	19
*FGFR2* only	10	4	14
*FGFR3* only	47	13	60
*FGFR1* + *FGFR2*	8	4	12
*FGFR1* + *FGFR3*	9	3	12
*FGFR2* + *FGFR3*	7	1	8
*FGFR1* + *FGFR2* + *FGFR3*	5	1	6
No *FGFR* alteration	53	10	63

Patients were classified as *FGFR*-altered if at least one reportable variant (any tier) was present in *FGFR1*, *FGFR2* or *FGFR3*. For gene-level analyses, each gene was coded as a separate binary variable (altered vs. not altered) irrespective of alterations in the other two genes, and all three genes were entered simultaneously in the multivariable model (Model B) so that each hazard ratio is adjusted for co-occurring alterations.

**Table 4:** Univariable Cox regression analysis of overall survival.

Variable	Overall Cohort (n = 194)		Non-Metastatic (n = 154)		Metastatic (n = 40)	
	HR (95% CI)	*p*	HR (95% CI)	*p*	HR (95% CI)	*p*
Metastatic disease	4.30 (2.90–6.38)	<0.001	–		–	
Age (per 10 years)	1.32 (1.12–1.54)	<0.001	1.40 (1.16–1.67)	<0.001	1.39 (0.94–2.06)	0.101
Male sex	0.82 (0.54–1.25)	0.350	0.84 (0.49–1.42)	0.513	2.00 (0.91–4.42)	0.085
ECOG PS 1 vs. 0	1.58 (0.97–2.56)	0.063	1.60 (0.93–2.75)	0.086	0.84 (0.28–2.55)	0.758
ECOG PS 2 vs. 0	2.38 (1.34–4.25)	0.003	2.25 (1.15–4.40)	0.017	1.46 (0.43–5.01)	0.547
Any comorbidity	1.11 (0.81–1.53)	0.507	1.05 (0.73–1.51)	0.792	1.14 (0.55–2.38)	0.723
Surgery + adjuvant CT ^†^	–		0.54 (0.34–0.87)	0.011	–	
Chemoradiotherapy ^†^	–		1.41 (0.93–2.14)	0.108	–	
No curative-intent therapy ^†^	–		1.21 (0.83–1.75)	0.317	–	
First-line systemic therapy received	–		–		0.26 (0.12–0.57)	<0.001
Any *FGFR* alteration	1.47 (1.04–2.08)	0.028	1.48 (1.00–2.19)	0.052	1.17 (0.56–2.45)	0.676
*FGFR1* alteration	1.65 (1.17–2.32)	0.004	1.72 (1.15–2.56)	0.008	1.71 (0.84–3.47)	0.137
*FGFR2* alteration	1.56 (1.08–2.25)	0.018	1.81 (1.18–2.76)	0.007	0.53 (0.24–1.17)	0.115
*FGFR3* alteration	1.06 (0.77–1.44)	0.723	1.03 (0.72–1.48)	0.859	1.46 (0.75–2.83)	0.261
*FGFR3* Tier I alteration	0.74 (0.48–1.12)	0.155	0.61 (0.36–1.02)	0.060	0.98 (0.46–2.08)	0.954
Any Tier I/II *FGFR* alteration	1.02 (0.73–1.43)	0.903	1.00 (0.68–1.48)	0.998	1.22 (0.61–2.43)	0.578

^†^ Reference: neoadjuvant chemotherapy followed by radical cystectomy (non-metastatic subgroup only). ECOG PS was available in 145 (overall), 115 (non-metastatic) and 30 (metastatic) patients. Wald *p* values are shown; likelihood-ratio *p* values for *FGFR* variables were: any *FGFR* 0.024/0.046/0.673; *FGFR1* 0.006/0.011/0.149; *FGFR2* 0.022/0.010/0.096; *FGFR3* 0.723/0.859/0.263 (overall/non-metastatic/metastatic). CI, confidence interval; ECOG PS, Eastern Cooperative Oncology Group performance status; HR, hazard ratio.

**Table 5:** Multivariable Cox regression analysis of overall survival.

Variable	HR (95% CI)	*p*
**Model A—overall cohort (n = 145; 119 deaths)**		
Any *FGFR* alteration	1.31 (0.87–1.97)	0.200
Metastatic disease	5.12 (3.15–8.33)	<0.001
ECOG PS 1 (vs. 0)	1.19 (0.72–1.97)	0.497
ECOG PS 2 (vs. 0)	1.42 (0.74–2.73)	0.294
Age (per 10 years)	1.35 (1.10–1.68)	0.005
**Model B—overall cohort (n = 145; 119 deaths)**		
*FGFR1* alteration	1.62 (1.07–2.44)	0.023
*FGFR2* alteration	1.05 (0.66–1.65)	0.841
*FGFR3* alteration	1.25 (0.86–1.81)	0.242
Metastatic disease	5.34 (3.23–8.86)	<0.001
ECOG PS 1 (vs. 0)	1.20 (0.72–2.00)	0.489
ECOG PS 2 (vs. 0)	1.48 (0.77–2.88)	0.242
Age (per 10 years)	1.32 (1.06–1.63)	0.012
**Model A—non-metastatic subgroup (n = 115; 89 deaths)**		
Any *FGFR* alteration	1.41 (0.88–2.26)	0.154
ECOG PS 1 (vs. 0)	1.13 (0.62–2.05)	0.689
ECOG PS 2 (vs. 0)	1.30 (0.60–2.83)	0.503
Surgery + adjuvant CT ^†^	0.47 (0.21–1.05)	0.065
Chemoradiotherapy ^†^	1.00 (0.53–1.88)	0.997
No curative-intent therapy ^†^	1.13 (0.62–2.07)	0.689
Age (per 10 years)	1.23 (0.92–1.63)	0.156
**Model B—non-metastatic subgroup (n = 115; 89 deaths)**		
*FGFR1* alteration	1.56 (0.95–2.57)	0.081
*FGFR2* alteration	1.56 (0.93–2.60)	0.091
*FGFR3* alteration	1.12 (0.71–1.76)	0.635
ECOG PS 1 (vs. 0)	1.03 (0.56–1.88)	0.933
ECOG PS 2 (vs. 0)	1.39 (0.63–3.06)	0.415
Surgery + adjuvant CT ^†^	0.43 (0.19–1.00)	0.050
Chemoradiotherapy ^†^	0.85 (0.43–1.68)	0.642
No curative-intent therapy ^†^	0.99 (0.53–1.86)	0.971
Age (per 10 years)	1.20 (0.90–1.59)	0.207
**Exploratory—metastatic subgroup (any *FGFR*) (n = 30; 30 deaths)**		
Any *FGFR* alteration	1.38 (0.51–3.77)	0.527
ECOG PS 1 (vs. 0)	0.69 (0.20–2.43)	0.568
ECOG PS 2 (vs. 0)	1.30 (0.36–4.69)	0.693
**Exploratory—metastatic subgroup (gene level) (n = 30; 30 deaths)**		
*FGFR1* alteration	2.18 (0.91–5.22)	0.081
*FGFR2* alteration	0.37 (0.14–1.00)	0.051
*FGFR3* alteration	1.49 (0.64–3.46)	0.353
ECOG PS 1 (vs. 0)	1.02 (0.31–3.39)	0.970
ECOG PS 2 (vs. 0)	1.56 (0.41–5.91)	0.509

Model A includes overall *FGFR* alteration status; Model B includes *FGFR1*, *FGFR2* and *FGFR3* alteration status simultaneously (overall *FGFR* status is not included, to avoid collinearity). Overall-cohort models are adjusted for metastatic status, ECOG PS and age (per 10 years); non-metastatic models for ECOG PS, primary treatment and age (^†^ reference: neoadjuvant chemotherapy + radical cystectomy); metastatic models for ECOG PS only and are exploratory given 30 events. Interaction between overall *FGFR* status and metastatic status (added to Model A, overall cohort): likelihood-ratio *p* = 0.59. Sensitivity analysis restricting *FGFR*-altered status to Tier I/II variants (overall cohort, adjusted as Model A): HR 1.36 (0.90–2.04), *p* = 0.141. Proportional hazards assumption was met for all models (scaled Schoenfeld residuals, all *p* > 0.05). Analyses were restricted to patients with available ECOG PS.

## Data Availability

The original contributions presented in this study are included in the article. Further inquiries can be directed to the corresponding author.
